# Diagnostic Accuracy of Magnetic Resonance Imaging in the Detection of Meniscal Injury in Patients With Knee Trauma: Keeping Arthroscopy as a Gold Standard

**DOI:** 10.7759/cureus.72343

**Published:** 2024-10-24

**Authors:** Danial Khalid, Junaid Iqbal, Khalid Mustafa, Raisa Altaf, Ramsha Fatima

**Affiliations:** 1 Radiology, Ziauddin University Hospital, Karachi, PAK; 2 Interventional Radiology, Liaquat National Hospital and Medical College, Karachi, PAK; 3 Radiology, Aga Khan University Hospital, Karachi, PAK; 4 Radiology, Liaquat National Hospital and Medical College, Karachi, PAK

**Keywords:** arthroscopy, magnetic resonance imaging (mri), meniscal tear, meniscus, non-invasive

## Abstract

Introduction: Damage to the knee's meniscal tissue is medically important because the meniscus plays a vital role in distributing weight, absorbing impact, and stabilizing the joint. Although arthroscopy continues to be the benchmark for the conclusive identification of knee-related injuries, magnetic resonance imaging (MRI) has gained traction as a widely used non-invasive technique for evaluating possible meniscal damage. The objective of this research is to assess the reliability of MRI as a diagnostic tool for identifying meniscal tears, with arthroscopic results serving as the definitive reference.

Methods: This observational study took place in the Radiology Department of Ziauddin University Hospital, located in Karachi, Pakistan, from June to November of 2019, subsequent to receiving ethical clearance from the hospital's review board. MRI assessments were conducted by seasoned senior radiologists who had a minimum of five years of domain-specific experience. Arthroscopic evaluations were executed by a skilled orthopedic surgeon. Information regarding patient demographics and clinical history was gathered through a pre-established protocol. Statistical analysis of the collected data was performed using SPSS Statistics version 21 (IBM Corp. Released 2012. IBM SPSS Statistics for Windows, Version 21.0. Armonk, NY: IBM Corp.).

Results: This research included a total of 141 participants, with 95 (67.4%) being male. The average age of the subjects was 38.89 years, with a standard deviation of 6.87 years. The mean body mass index (BMI) stood at 22.81 kg/m^2 with a standard deviation of 1.81. The typical duration since the injury occurred was 3.23 months, with a standard deviation of 1.6 months. According to the MRI evaluations, 83 participants (58.9%) exhibited a tear in the medial meniscus, while 58 (41.1%) showed a tear in the lateral meniscus. The MRI tests revealed a sensitivity of 94% and a specificity of 87.8% in identifying meniscal tears, boasting an overall diagnostic precision of 92.2%.

Conclusion: This study substantiates that MRI possesses a high degree of sensitivity in the identification of meniscal injuries. It serves as an effective preliminary assessment tool for pinpointing patients who could potentially benefit from therapeutic arthroscopy, thereby often negating the necessity for diagnostic arthroscopy.

## Introduction

The lateral and medial menisci, crescent-like fibrocartilaginous entities that encompass nearly 70% of the tibial plateau's articulating surface, mainly function in the dissemination of mechanical force and dampening of impact within the tibiofemoral joint [1]. Owing to the meniscus's essential roles in weight distribution, impact absorption, and joint stability, damage to this structure carries substantial clinical ramifications [2].

Isolated tears in the meniscus frequently arise when rotational or shearing forces act upon the tibiofemoral joint, particularly when there's an increased axial stress on the menisci. Such scenarios include carrying heavy loads, abrupt starts and stops, directional changes, leaping, and postures that involve high levels of closed kinetic chain flexion, such as kneeling or squatting [2]. The estimated incidence rate of meniscal tears stands at approximately 60 per 100,000 individuals, and the occurrence of these injuries is on the rise, largely due to heightened sports engagement and enhanced diagnostic techniques [3].

Torn meniscal or ligamentous structures within the knee can result in intense pain and functional impairment, necessitating immediate medical intervention and management. Injuries to the meniscus can have severe repercussions, particularly when sustained during sports activities, emphasizing the need for prompt and precise diagnosis [4]. The initial clinical evaluation and diagnostic procedures are pivotal in deciding whether surgical intervention is required following a meniscal injury. A comprehensive examination should encompass an in-depth injury history, tactile assessment of the affected area, and specialized diagnostic tests [5].

Although arthroscopy is regarded as the gold standard for diagnosing internal knee abnormalities, it also functions as a minimally invasive surgical technique for addressing intra-articular conditions [4]. Nonetheless, arthroscopic procedures come with inherent risks, including life-threatening complications like pulmonary embolism, warranting cautious and well-informed application [4].

Despite initial skepticism nearly three decades ago regarding its therapeutic value and cost-efficiency in treating knee conditions, magnetic resonance imaging (MRI) has earned its place as the leading non-invasive diagnostic instrument, extensively employed for assessing intra-articular knee lesions [6]. Over the years, research has concentrated on evaluating the diagnostic accuracy of MRI against knee arthroscopy, considered the "gold standard." Nonetheless, there is ongoing discussion about the dependability of MRI in identifying articular cartilage defects in the knee. Sensitivity rates have been cited to vary widely, from a low of 15% to a high of 60%, contingent upon the depth of the lesion [7,8]. In Pakistan, multiple studies have yielded conflicting results. For instance, studies by Arif et al. [9] and Anwar et al. [10] found MRI to be reliable for diagnosing meniscal tears, whereas Sajid et al. [11] argued that MRI could serve as a preliminary screening tool, but arthroscopy remains the ultimate diagnostic method. In light of these varying results, this research aims to augment the current body of knowledge by evaluating the effectiveness of MRI for the diagnosis of meniscal injuries, as compared to arthroscopy, within our medical institution.

## Materials and methods

This observational study was carried out in the Radiology Department of Ziauddin University Hospital in Karachi, Pakistan, between June and November 2019, following ethical clearance from the Ethical Review Board of Ziauddin University Hospital (approval number: 2020-8891-25431). The research encompassed participants aged between 18 and 50 years, of any gender, who were clinically suspected to have meniscal injuries. These individuals underwent an initial physical examination, followed by MRI and arthroscopic evaluations. Exclusion criteria included a previous history of fractures, knee surgeries, degenerative joint disorders, inflammatory conditions, recent injuries (more than four weeks old), or multiple ligament injuries in the knee. Based on a sensitivity rate of 92% and a specificity rate of 75% [10], along with a 44.4% prevalence rate of knee meniscal injuries [12], the estimated sample size was set at 131 participants, with a 95% confidence level and a 7% margin of error.

MRI procedures were conducted using a 1.5 Tesla Magneton Harmony (SIEMENS, Munich, Germany) scanner equipped with a specialized knee coil. The imaging followed a multiplanar, multisequential protocol in accordance with departmental guidelines, incorporating T1, T2, short T1 inversion recovery, and proton density-weighted images across different planes. The review of MRI scans was overseen by expert senior radiologists with no less than five years of specialized experience following their fellowship. Arthroscopic examinations were executed by a capable orthopedic surgeon. A pre-defined template was used to capture a range of patient details, including name, age, sex, hospital ID number, height, weight, body mass index (BMI), the time that has passed since the knee injury, and the presence or absence of meniscal issues as confirmed by both MRI and arthroscopy.

The collection and evaluation of data were conducted using SPSS Statistics version 21 (IBM Corp. Released 2012. IBM SPSS Statistics for Windows, Version 21.0. Armonk, NY: IBM Corp.). For numerical variables, mean values and standard deviations were calculated, while for categorical variables, frequencies and percentages were tabulated. A 2 x 2 table was formulated to ascertain the sensitivity, specificity, positive and negative predictive values, and the overall diagnostic accuracy of MRI in identifying meniscal injuries, using arthroscopic findings as the definitive standard. Cohen's kappa statistic was employed to assess the concordance between MRI and arthroscopy results. A p-value of ≤0.05 was deemed statistically significant.

## Results

This research involved a total of 141 participants, with 95 (67.4%) being male. The average age of the subjects was 38.89 years, with a standard deviation of 6.87 years. The BMI stood at 22.81 kg/m^2 with a standard deviation of 1.81. The typical duration since the injury occurred was 3.23 months, with a standard deviation of 1.6 months. According to the MRI evaluations, 83 participants (58.9%) exhibited a tear in the medial meniscus, while 58 (41.1%) showed a tear in the lateral meniscus. Table 1 provides a breakdown of the frequency, location, and types of injuries among medial and lateral meniscal tears.

**Table 1:** Frequency of location and type of injury among medial and lateral meniscal tears

Location	Medial		Lateral	
	Frequency	%	Frequency	%
Anterior horn	4	4.8	4	6.9
Body	9	10.8	14	24.1
Posterior horn	32	38.6	19	32.8
≥2 compartment	38	45.8	21	36.2
Type				
Longitudinal	29	34.9	18	31.0
Horizontal	3	3.6	6	10.3
Radial	4	4.8	12	20.7
Vertical flap	1	1.2	0	0.0
Horizontal flap	2	2.4	0	0.0
Complex	44	53.0	22	37.9

Table 2 provides a comparison of MRI and arthroscopy findings for medial and lateral menisci.

**Table 2:** Comparison of MRI findings with arthroscopy findings MRI: magnetic resonance imaging

MRI finding	Arthroscopy findings		Total
	Yes n (%)	No n (%)	
Medial injuries			
Yes	56 (94.9)	3 (5.0)	59 (100)
No	3 (12.5)	21 (87.5)	24 (100)
Total	59 (71.0)	24 (28.9)	83 (100)
Lateral injuries			
Yes	38 (92.6)	3 (7.3)	41 (100)
No	2 (11.7)	15 (88.2)	17 (100)
Total	40 (68.9)	18 (31.0)	58 (100)

MRI assessments yielded 94 true positives, six false positives, five false negatives, and 36 true negatives. These figures translated to a sensitivity of 94.9% and a specificity of 85.7% for MRI. Employing arthroscopy as the ultimate standard for evaluation, the MRI exhibited a predictive positive value of 94%, a predictive negative value of 87.8%, and an aggregate diagnostic precision of 92.2%, as shown in Table 3.

**Table 3:** Diagnostic precision for overall, medial, and lateral meniscal injuries

Diagnostic accuracy measures	Overall	Medial	lateral
Sensitivity (%)	94.9	94.9	95.0
Specificity (%)	85.7	87.5	83.3
Positive predictive value (%)	94	94.9	92.6
Negative predictive value (%)	87.8	87.5	88.2
Diagnostic accuracy (%)	92.2	92.7	91.3

## Discussion

Once considered merely vestigial remnants from embryonic development, the menisci are now recognized as essential components for maintaining normal knee function and long-term joint health [13,14]. MRI, being a non-invasive and radiation-free technique, offers distinct advantages for evaluating soft tissues. Although arthroscopy is invasive and carries associated risks, its diagnostic accuracy varies between 64% and 94% [15]. The primary objective of this study was to juxtapose MRI and arthroscopy in the assessment of knee injuries.

In the current study, MRI identified a higher number of medial meniscal injuries in contrast to lateral ones, aligning with previous Pakistani research [10,11]. Numerous studies from outside Pakistan also report a greater incidence of medial versus lateral injuries [2,16,17]. Orthopedic surgeons often observe anecdotally that lateral meniscal tears are frequently observed in younger populations, whereas medial tears are more prevalent in older age groups [17]. The medial meniscus has more robust capsular attachments and covers around half of the medial tibial plateau, while the smaller lateral meniscus covers around 70% of the lateral plateau [18]. The posterior lateral meniscus is weakly attached through popliteomeniscal fascicles. Reported dimensions for the medial meniscus range from measurements that span from 40.5 to 45.5 mm in length and are 27 mm in width, compared to the dimensions for the lateral meniscus, which range from 32.4 to 35.7 mm in length and 26.6 to 29.3 mm in width [19,20]. These anatomical and biomechanical distinctions likely contribute to the higher frequency of isolated lateral tears in individuals under 20 years of age. The lateral meniscus tends to be more pliable, possesses looser capsular connections, and encompasses a greater area of the tibial plateau. As a result, isolated lateral meniscal injuries are more likely to happen after traumatic events in younger people.

In this research, MRI demonstrated an overall diagnostic accuracy of 92.2% when compared to arthroscopy, featuring a sensitivity of 94.9% and a specificity of 85.7%. The comprehensive performance of MRI in terms of both sensitivity and specificity surpassed that of earlier studies, suggesting its viability as a reliable diagnostic instrument for meniscal tears [2,16,11,21]. Additionally, the concordance between MRI and arthroscopic findings was nearly flawless in this investigation. However, other studies have reported lower kappa values. For example, the study by Anderson et al. revealed that the interobserver consistency in using MRI to precisely identify the nature and site of tears varied from moderate to significant, exhibiting kappa coefficients ranging between 0.46 and 0.72 [22]. Dunn et al. indicated that the agreement between MRI and arthroscopy for categorizing the type and location of tears was moderate to good, with kappa values of 0.61-0.63 [23]. Another study that employed the ISAKOS classification system reported moderate agreement between MRI and arthroscopy for meniscal tears [24]. The elevated diagnostic accuracy and agreement in this study are likely attributable to the exclusion of patients with anterior cruciate ligament (ACL) injuries. Kim et al. noted reduced diagnostic values and agreement in cases involving ACL injuries [2]. Hence, in the absence of ACL injuries, MRI appears to be a dependable method for detecting the presence of meniscal tears, although it may not be as reliable for the detailed classification of tear types and locations based on current ISAKOS guidelines.

This study does have notable limitations, such as a restricted sample size drawn from a single institution. Due to the small size of subgroups, the diagnostic accuracy was not assessed based on the location and type of tears. Furthermore, patients with ACL injuries were deliberately excluded. Given these constraints, generalizing the study's findings to all types and locations of meniscal tears would be imprudent. Future research with a more extensive sample size could fill the existing gaps and validate the conclusions of this study. Such an investigation should assess diagnostic accuracy by tear type and location and should also include patients with ACL injuries. The limitation of being a single-center study could be mitigated through a multi-institutional approach.

## Conclusions

This study affirms the high sensitivity of MRI in identifying meniscal tears. It serves as an effective preliminary assessment tool for pinpointing patients who could potentially benefit from therapeutic arthroscopy, thereby often negating the necessity for diagnostic arthroscopy in many cases.
